# Validity and reliability of the Turkish version of the Multidimensional Dyspnea Profile in outpatients with respiratory disease

**DOI:** 10.3906/sag-2001-155

**Published:** 2020-12-17

**Authors:** Buse ÖZCAN KAHRAMAN, Turhan KAHRAMAN, İsmail ÖZSOY, Aylin TANRIVERDİ, Aslı PAPURCU, Nazenin Hande SEZGİN, Karya POLAT, Serap ACAR, Aylin ÖZGEN ALPAYDIN, Can SEVİNÇ, Sema SAVCI

**Affiliations:** 1 Department of Cardiopulmonary Physiotherapy-Rehabilitation, School of Physical Therapy and Rehabilitation,Dokuz Eylül University, İzmir Turkey; 2 Department of Physiotherapy and Rehabilitation, Faculty of Health Sciences, İzmir Kâtip Çelebi University, İzmir Turkey; 3 Department of Physiotherapy and Rehabilitation, Faculty of Health Sciences, Selçuk University, Konya Turkey; 4 Department of Physical Therapy and Rehabilitation, Graduate School of Health Sciences, Dokuz Eylül University, İzmir Turkey; 5 Department of Chest Disease, Faculty of Medicine, Dokuz Eylül University, İzmir Turkey

**Keywords:** Validity, reliability, dyspnea, multidimensional, Turkish, questionnaire

## Abstract

**Background/aim:**

Dyspnea is the subjective feeling of breathing discomfort, which is a significant problem for patients with heart and respiratory disease and also an important determinant of exercise tolerance, quality of life, and mortality in various diseases. Most of the scales are not enough to investigate the multidimensional effects of dyspnea; therefore, the Multidimensional Dyspnea Profile (MDP) was developed and validated in many languages. This study aimed to translate and culturally adapt the MDP into Turkish and investigate the psychometric properties of this adapted version in outpatients with respiratory disease.

**Materials and methods:**

The MDP was translated and culturally adapted into Turkish following published guidelines. A total of 170 outpatients with respiratory disease were included to assess psychometric properties. The factorial structure was investigated using a principal component analysis. Two situations were used in this study evaluating dyspnea in activity-related and resting conditions. We formulated 17 hypotheses for each MDP domain (in total 68) to assess construct validity, and correlations were investigated between the MDP and measures of body mass index, pulmonary function test, other dyspnea assessments, anxiety, depression, and health-related quality of life. To investigate the test-retest reliability, the MDP was administered again after 1-h and 1 week.

**Results:**

Internal consistency of the MDP was excellent (Cronbach’s alpha coefficients ranged from 0.89 to 0.93). The exploratory factor analysis revealed 2 components explaining a 70% and 76% variance. Overall, 64 of the 68 predetermined hypotheses (94%) were confirmed to test construct validity. The MDP showed excellent test-retest reliability for a 1-hperiod (intraclass correlation coefficient values ranged from 0.98 to 0.99). However, test-retest reliability decreased moderate-to-high after 1 week (0.53–0.80).

**Conclusion:**

The MDP was successfully translated and culturally adapted into Turkish and this version showed good psychometric properties including the factorial structure, internal consistency, test-retest reliability, and construct validity to assess multidimensional aspects of dyspnea.

## 1. Introduction

Dyspnea is the subjective feeling of breathing discomfort, which is a significant problem for patients with heart and respiratory disease [1]. Dyspnea is also an important determinant of exercise tolerance, quality of life, and mortality in various diseases [2]. Recent research showed that dyspnea is multidimensional, and different afferent mechanisms can cause these dimensional variations [1–4].

Most of the common dyspnea measurements do not adequately assess the complexity of dyspnea [5]. Scales such as the Visual Analogue Scale (VAS) and the Modified Borg Scale (MBS) are used to measure the severity of dyspnea in unidimensional [6,7], or the Baseline/ Transitional Dyspnea Index (BDI/TDI) and the modified Medical Research Council (mMRC) Scale are used to assess the effects of dyspnea on exercise capacity [8]. Unidimensional scales are specific for a time point (current or recalled) but do not evaluate the quality of unpleasantness, breathing discomfort, or related emotional experiences. Therefore, these scales are not enough to investigate the multidimensional effects of dyspnea in individuals with chronic pulmonary disease.

The Dyspnea-12 score [9], Cancer Dyspnea Scale [10], and the Multidimensional Dyspnea Profile (MDP) [4,5,11] scales are multidimensional tools to assess dyspnea. The Dyspnea-12 gives a sum score of total items and does not assess feelings related to chest constriction, concentration, anxiety, fear, or frustration. However, the MDP presents sensory qualities, discomfort, and emotional responses to the dyspnea experience [12]. The Cancer Dyspnea Scale also gives a sum score, except for an anxiety score, and does not provide enough information about other dimensions of dyspnea [10]. The MDP can be used in clinical settings [5] and experimental settings [4]. Users can define a specified time frame or situation for the measure, which can be changed based on the study design. It is easy to understand the MDP, and the administration of MDP takes approximately 2 min for most subjects and patients [12].

The MDP has an increasing use in international studies and was previously validated and/or translated into Dutch for Belgium and Netherlands; English for the USA, Canada, UK, and Australia; French for Belgium, Canada, and France; German for Germany, Swedish for Sweden, and Portuguese for Brazil [12–18]. However, the MDP does not have a Turkish validation. Therefore, this study aimed to translate and culturally adapt the MDP into Turkish investigating its psychometric properties including the factorial structure, internal consistency, test-retest reliability, and construct validity.

## 2. Materials and methods

### 2.1. Design and participants

Patients above the age of 18 years who presented to the Department of Chest Diseases, Dokuz Eylül University Hospital with documented physician-diagnosed chronic respiratory disease, were recruited to this validation study from July to December 2018. Patients with acute coronary syndrome, advanced or metastatic cancer or the inability to speak or understand Turkish were excluded.

Despite the lack of an internationally accepted consensus about the minimum required sample size for validation studies, it is generally recommended to include 2–20 participants per item [19]. Therefore, we determined an a priori sample size of 110 patients, for 10 participants per item.

The study was approved by the Noninvasive Research Ethics Board of Dokuz Eylül University Hospital (approval number: 2018/18-40 and date: 19.07.2018) and performed following the ethical standards as laid down in the 1964 Declaration of Helsinki (as revised in Brazil 2013). All the participants gave written informed consent before participation in the study.

### 2.2. Study protocol

To examine the intrarater reliability, 1 physiotherapist evaluated the same patient after 1-h and 1 week using the MDP. To assess construct validity, the spirometry results, the mMRC scale, VAS, MBS, Hospital Anxiety and Depression Scale (HADS), and Nottingham Health Profile (NHP) were used.

### 2.3. Translation and crosscultural adaptation

Permission for the Turkish validation study was obtained by the Mapi Research Trust, Lyon, France. The MDP was translated and culturally adapted following the published guidelines [20]. First, English-to-Turkish translations were done by 2 translators. After that, the expert committee compared and discussed the 2 versions and made them 1 form for the Turkish-translated version. Two translators performed back-translation of this from Turkish to English. These translations were reviewed and compared with the original scale by the expert committee. The back-translation was sent to the Mapi Research Trust and it was approved. Ten patients were tested using the prefinal MDP version, and they did not suggest any change for clarity, wording, terminology, or instructions. After this, the final Turkish version of the MDP was ready to use.

### 2.4. Outcome measures

The demographic and clinical characteristics of the patients were collected from the latest medical records while crosschecking with patient interviews.

The MDP contains 11 items that evaluate Immediate Perception (IP) (6 items; total score range is 0–60) and Emotional Response (ER) (score range is 0–50) domains of dyspnea [12]. The items are measured on a rating scale of 0–10, with higher scores indicating greater intensity, unpleasantness, or distress. Each item can be calculated separately, or domain scores can be given for IP and ER scores. The MDP can be completed in 2–3 min. The focal period for the MDP is determined by users as appropriate for the intent of the research or clinical situation (e.g., “right now” or “at the end of a minute of a particular activity”). In this study, the time frame was “the past 2 weeks” and the situation was “while resting without doing any physical activity” named as the MDP-Resting and “at the end of climbing a flight of 2-floor stairs” named as the MDP-Activity. The original MDP and its translations including Turkish are distributed by the Mapi Research Trust. For permission to use the Turkish version of the MDP, please access the website of the Mapi Research Trust (u2920).

Other dyspnea instruments included the mMRC [21], VAS [22], and MBS [23]. The VAS and MBS were administered in the same way as the MDP (i.e., “while resting without doing any physical activity during the past 2 weeks” was asked in the VAS-Rest and MBS-Rest, and “at the end of climbing a flight of 2-floor stairs during the past 2 weeks” was asked in the VAS-Activity and MBS-Activity).

The HADS was used to assess anxiety and depression [24]. The health-related quality of life was measured using the NHP which includes 6 subdomains: energy level, pain, emotional reactions, sleep, social isolation, and physical abilities [25].

### 2.5. Statistical analysis

The statistical analysis was performed using IBM SPSS software (Version 25.0, IBM Corp., Armonk, NY). The patients’ demographic characteristics and assessment results were described using descriptive statistics.

The Cronbach’s alpha coefficients were calculated to assess the internal consistency. The Cronbach’s alpha coefficients were interpreted as excellent, >0.80; adequate, 0.70–0.79; and inadequate, <0.70 [26]. The intraclass correlation coefficient (ICC) values with 95% confidence intervals (95% CI) were calculated to assess test-retest reliability. The ICC values were reported as very low ≤0.25, low = 0.26–0.49, moderate = 0.50–0.69, high = 0.70–0.89, and very high ≥0.90 [27].

An exploratory principal components analysis using varimax rotation, using baseline data to determine the underlying factorial structure of the MDP was used to verify the 2-factor structure as defined in the original validation study [12].

To assess the construct validity, correlations were calculated between the MDP and measures of body mass index, pulmonary function test, other dyspnea assessments (VAS, mMRC Scale, and MBS), anxiety, depression, and health-related quality of life. We formulated 17 hypotheses for each MDP domain (in total, 34 for MDP-Activity, and 34 for MDP-Resting) on the strength of the association of the MDP and construct variables. According to previous validation studies to investigate the divergent validity, we expected negligible/nonsignificant or weak correlations with body mass index (BMI) and pulmonary function test results. To assess concurrent validity, we expected that higher MDP-Activity scores were associated with higher VAS and MBS activity scores. Similarly, higher MDP-Resting scores were expected to be associated with higher VAS and MBS resting scores. Moderate-to-strong correlations were expected between MDP and anxiety, depression, and health-related quality of life (for the details, see online supplementary material). A correlation coefficient of <0.1 was considered negligible; 0.10–0.30 was considered small, 0.30–0.50 moderate, 0.50–0.70 strong, and 0.70–0.90 very strong. The level of significance was set as P < 0.05 in all analyses.

## 3. Results

In total, 186 patients were screened and 170 of them met inclusion criteria and completed the baseline MDP, and 30 patients completed MDP again after 1 h, and 89 patients completed the MDP again after 1 week. The mean age of the participants was 61 (SD = 14) years. There were 52 female (30.6%) and 118 male (69.4%) participants. Diagnoses were chronic obstructive pulmonary disease (COPD), (46.5%), asthma (22.9%), and miscellaneous (30.6%) such as pneumonia, idiopathic pulmonary fibrosis, etc. The demographics and clinical characteristics of the participants are presented in Table 1.

**Table 1:** Demographic and clinical characteristics of the participants (n = 170).

	Nonmissing observations	Mean (SD)
Age (years)	170	61 (14)
Sex, n (%)	170	
Female		52 (30.6)
Male		118 (69.4)
BMI, kg/m2	170	27.3 (4.9)
Diagnosis, n (%)	170	
COPD		79 (46.5)
Asthma		39 (22.9)
Miscellaneous (pneumonia, idiopathic pulmonary fibrosis, etc.)		52 (30.6)
FEV1/FVC % predicted	131	67.8 (13.7)
FEV1 % predicted	136	64.7 (22.9)
FVC % predicted	138	75.5 (19.9)
MDP-Activity, Immediate Perception	170	26.4 (17.6)
MDP-Activity, Emotional Response	170	13.4 (15.4)
MDP-Resting, Immediate Perception	170	6.3 (10.1)
MDP-Resting, Emotional Response	170	6.9 (10.8)
VAS – Resting, mm	170	12.0 (21.5)
VAS – Activity, mm	170	49.8 (34.2)
MBS – Resting	170	1.4 (2.0)
MBS – Activity	170	5.3 (2.8)
mMRC Scale	170	2.2 (1.2)
HADS – Anxiety	170	6.5 (5.3)
HADS – Depression	170	5.8 (4.6)
NHP – Energy level	170	40.5 (37.0)
NHP – Pain	170	22.4 (29.9)
NHP – Emotional reaction	170	21.9 (26.8)
NHP – Social isolation	170	17.1 (25.1)
NHP – Sleep	170	20.9 (23.1)
NHP – Physical abilities	170	25.5 (25.7)

BMI: body mass index; COPD: chronic obstructive pulmonary disease; MDP: Multidimensional Dyspnea Profile; VAS: Visual Analogue Scale; MBS: Modified Borg Scale; mMRC: Modified Medical Research Council; HADS: Anxiety and Depression Scale; NHP: Nottingham Health Profile.

Internal consistency of the MDP-Activity IP and ER domains was excellent (Cronbach’s alpha coefficient = 0.92 and 0.93, respectively). Internal consistency of the MDP-Resting IP and ER was excellent (Cronbach’s alpha coefficient = 0.90 and 0.89, respectively).

Test-retest ICC values between recall ratings for the approximate 1-h interval ranged from 0.80 to 0.99 for the individual items of the 2 domains of MDP-Activity. Both the MDP-Activity IP and ER had 0.99 of ICC after the 1-h interval. Test-retest ICCs between recall ratings for the approximate 1-week interval were lower ranging from 0.38 to 0.62 for the individual items the 2 domains of MDP-Activity. The ICC values for the MDP-Activity IP and ER after the 1-week interval were 0.80 and 0.77, respectively. The ICC values of the MDP-Resting were lower. Individual item ICC values of the 2 domains of the MDP-Resting ranged from 0.57 to 0.99. The ICC values for both the MDP-Resting IP and ER after the 1-h interval were 0.98. The ICC values for the MDP-Resting IP and ER after the 1-week interval were 0.53 and 0.54, respectively. Table 2 presents detailed test-retest reliability results.

**Table 2:** Factor loadings and test-retest reliability results of the MDP-Activity and MDP-Resting.

		MDP-Activity					MDP-Resting			
		Baseline –After 1-h(n = 170–30)		Baseline –After 1 week(n = 170–89)			Baseline –After 1-h(n = 170–30)		Baseline –After 1 week(n = 170–89)	
	Factorsloadings	ICC	95% CI	ICC	95% CI	Factors loadings	ICC	95% CI	ICC	95% CI
Immediate Perception items										
1. Intensity	0.86	0.93	0.86–0.97	0.61	0.47–0.73	0.73	0.91	0.82–0.96	0.38	0.19–0.54
2. Muscle work/Effort	0.86	0.98	0.96–0.99	0.59	0.44–0.71	0.86	0.95	0.91–0.98	0.21	0.01–0.40
3. Not enough air/Smothering/Air hunger	0.80	0.95	0.90–0.98	0.56	0.40–0.69	0.81	0.87	0.75–0.94	0.37	0.17–0.53
4. Tight/Constricted	0.83	0.85	0.70–0.92	0.52	0.36–0.66	0.76	0.98	0.95–0.99	0.21	0.01–0.40
5. Mental effort/Concentration	0.57	0.80	0.63–0.90	0.62	0.47–0.73	0.75	0.57	0.26–0.77	0.42	0.24–0.58
6. Breathing a lot(rapid, deep, heavy)	0.86	0.89	0.77–0.94	0.58	0.42–0.70	0.72	0.99	0.99–0.99	0.12	-0.09–0.32
Immediate Perception Domain (Mean of 6 items)	–	0.99	0.99–0.99	0.80	0.69–0.87	–	0.98	0.95–0.99	0.53	0.28–0.69
Emotional Response items										
1. Depressed	0.87	0.98	0.97–0.99	0.50	0.32–0.64	0.83	0.96	0.92–0.98	0.21	0.01–0.39
2. Anxious	0.86	0.99	0.98–0.99	0.59	0.43–0.71	0.86	0.92	0.84–0.96	0.21	0.01–0.40
3. Frustrated	0.88	0.96	0.92–0.98	0.56	0.40–0.68	0.83	0.95	0.90–0.98	0.27	0.07–0.45
4. Angry	0.78	0.98	0.96–0.99	0.38	0.19–0.55	0.70	0.95	0.89–0.97	0.05	-0.15–0.26
5. Afraid	0.82	0.99	0.97–0.99	0.62	0.48–0.73	0.75	0.88	0.76–0.94	0.26	0.06–0.44
Emotional Response Domain (Mean of 5 items)	–	0.99	0.99–0.99	0.77	0.65–0.85	–	0.98	0.95–0.99	0.54	0.27–0.70

MDP: Multidimensional Dyspnea Profile; ICC: Intraclass Correlation Coefficient; CI: Confidence Interval.

The exploratory factor analysis revealed 2 components explaining 76% variance for MDP-Activity and 70% variance for MDP-Resting (Table 2). The Kaiser–Meyer–Olkin (KMO) Measure of Sampling Adequacy and Bartlett’s Test of Sphericity (P < 0.001) results showed that the respondent data for factor analysis was suitable for MDP (KMO values were 0.898 for MDP-Activity and 0.903 for MDP-Resting).

All predetermined hypotheses were confirmed for MDP-Activity IP and 16 of the 17 hypotheses (94%) were confirmed for the ER domain. All predetermined hypotheses were confirmed for the MDP-Resting IP and this ratio was 82% for the MDP-Resting ER domain. Overall, 64 of the 68 hypotheses (94%) were confirmed (Table 3, Table 4, Table 5, and Table 6). The associations were in the expected direction in that higher MDP-Activity scores were associated with higher VAS and MBS activity scores (Table 3, Table 4, and Table 7). Similarly, higher MDP-Resting scores were associated with higher VAS and MBS resting scores (Table 5, Table 6, and Table 7). The MDP-Activity and Resting scores were significantly correlated with anxiety, depression, and health-related quality of life scores. As expected, negligible/nonsignificant correlations with BMI were overserved. We also expected nonsignificant or weak correlations with pulmonary function test results, and all hypotheses were confirmed. Correlations between the MDP–Activity, and MDP–Resting domains and the other variables are presented in Table 7.

**Table 3:** Hypothesis testing to assess the construct validity of the MDP-Activity, Immediate Perception.

Hypothesis	Construct validity is confirmed when the correlation is	Results	Interpretation of results
1. Negligible/nonsignificant correlation withBMI	Negligible/nonsignificant(|rho| ≤ 0.1; P > 0.05)	0.02	Confirmed
2. Nonsignificant/weak correlation withFEV1/FVC % predicted	Nonsignificant (P > 0.05) or Weak(0.1 ≤ |rho| ≤ 0.3)	–0.03	Confirmed
3. Nonsignificant/weak correlation withFEV1 % predicted	Nonsignificant (P > 0.05) or Weak(0.1 ≤ |rho| ≤ 0.3)	–0.19	Confirmed
4. Nonsignificant/weak correlation withFVC % predicted	Nonsignificant (P > 0.05) or Weak(0.1 ≤ |rho| ≤ 0.3)	–0.24	Confirmed
5. Moderate correlation with VAS – Resting	Moderate (0.3 ≤ |rho| ≤ 0.5)	0.48	Confirmed
6. Strong correlation with VAS – Activity	Strong (|rho| ≥ 0.5)	0.68	Confirmed
7. mMRC Scale	Moderate (0.3 ≤ |rho| ≤ 0.5)	0.38	Confirmed
8. MBS – Resting	Moderate (0.3 ≤ |rho| ≤ 0.5)	0.41	Confirmed
9. MBS – Activity	Strong (|rho| ≥ 0.5)	0.59	Confirmed
10. HADS – Anxiety	Moderate (0.3 ≤ |rho| ≤ 0.5)	0.37	Confirmed
11. HADS – Depression	Weak (0.1 ≤ |rho| ≤ 0.3)	0.26	Confirmed
12. NHP – Energy level	Moderate (0.3 ≤ |rho| ≤ 0.5)	0.43	Confirmed
13. NHP – Pain	Moderate (0.3 ≤ |rho| ≤ 0.5)	0.44	Confirmed
14. NHP – Emotional reaction	Moderate (0.3 ≤ |rho| ≤ 0.5)	0.37	Confirmed
15. NHP – Social isolation	Moderate (0.3 ≤ |rho| ≤ 0.5)	0.37	Confirmed
16. NHP – Sleep	Moderate (0.3 ≤ |rho| ≤ 0.5)	0.37	Confirmed
17. NHP – Physical abilities	Moderate (0.3 ≤ |rho| ≤ 0.5)	0.51	Confirmed

Statistically significant correlations are shown in bold. Confirmed hypotheses for the MDP-Activity, Immediate Perception = 17/17 (100%). MDP: Multidimensional Dyspnea Profile; VAS: Visual Analogue Scale; mMRC: Modified Medical Research Council; HADS: Hospital Anxiety and Depression Scale; BMI: body mass index; MBS: Modified Borg Scale; NHP: Nottingham Health Profile.

**Table 4:** Hypothesis testing to assess the construct validity of the MDP-Activity, Emotional Response.

Hypothesis	Construct validity is confirmed when the correlation is	Results	Interpretation of results
1. BMI	Negligible/nonsignificant(|rho| ≤ 0.1; P > 0.05)	0.13	Confirmed
2. FEV1/FVC % predicted	Nonsignificant (P > 0.05) or Weak(0.1 ≤ |rho| ≤ 0.3)	0.09	Confirmed
3. FEV1 % predicted	Nonsignificant (P > 0.05) or Weak(0.1 ≤ |rho| ≤ 0.3)	–0.09	Confirmed
4. FVC % predicted	Nonsignificant (P > 0.05) or Weak(0.1 ≤ |rho| ≤ 0.3)	–0.18	Confirmed
5. VAS – Resting	Moderate (0.3 ≤ |rho| ≤ 0.5)	0.36	Confirmed
6. VAS – Activity	Strong (|rho| ≥ 0.5)	0.59	Confirmed
7. mMRC Scale	Moderate (0.3 ≤ |rho| ≤ 0.5)	0.30	Confirmed
8. MBS – Resting	Moderate (0.3 ≤ |rho| ≤ 0.5)	0.35	Confirmed
9. MBS – Activity	Strong (|rho| ≥ 0.5)	0.39	Not confirmed
10. HADS – Anxiety	Moderate to strong (|rho| ≥ 0.3)	0.53	Confirmed
11. HADS – Depression	Moderate to strong (|rho| ≥ 0.3)	0.34	Confirmed
12. NHP – Energy level	Moderate (0.3 ≤ |rho| ≤ 0.5)	0.42	Confirmed
13. NHP – Pain	Moderate (0.3 ≤ |rho| ≤ 0.5)	0.44	Confirmed
14. NHP – Emotional reaction	Moderate (0.3 ≤ |rho| ≤ 0.5)	0.50	Confirmed
15. NHP – Social isolation	Moderate (0.3 ≤ |rho| ≤ 0.5)	0.42	Confirmed
16. NHP – Sleep	Moderate (0.3 ≤ |rho| ≤ 0.5)	0.34	Confirmed
17. NHP – Physical abilities	Moderate (0.3 ≤ |rho| ≤ 0.5)	0.50	Confirmed

Statistically significant correlations are shown in bold. Confirmed hypotheses for the MDP-Activity, Emotional Response = 16/17 (94%). MDP: Multidimensional Dyspnea Profile; VAS: Visual Analogue Scale; BMI: body mass index; MBS: Modified Borg Scale; mMRC: Modified Medical Research Council; HADS: Hospital Anxiety and Depression Scale; NHP: Nottingham Health Profile.

**Table 5:** Hypothesis testing to assess the construct validity of the MDP-Resting, Immediate Perception.

Hypothesis	Construct validity is confirmed when the correlation is	Results	Interpretation of results
1. BMI	Negligible/nonsignificant(|rho| ≤ 0.1; P > 0.05)	0.01	Confirmed
2. FEV1/FVC % predicted	Nonsignificant (P > 0.05) or Weak(0.1 ≤ |rho| ≤ 0.3)	0.19	Confirmed
3. FEV1 % predicted	Nonsignificant (P > 0.05) or Weak(0.1 ≤ |rho| ≤ 0.3)	–0.03	Confirmed
4. FVC % predicted	Nonsignificant (P > 0.05) or Weak(0.1 ≤ |rho| ≤ 0.3)	–0.06	Confirmed
5. VAS – Resting	Strong (|rho| ≥ 0.5)	0.75	Confirmed
6. VAS – Activity	Moderate (0.3 ≤ |rho| ≤ 0.5)	0.48	Confirmed
7. mMRC Scale	Moderate (0.3 ≤ |rho| ≤ 0.5)	0.31	Confirmed
8. MBS – Resting	Strong (|rho| ≥ 0.5)	0.60	Confirmed
9. MBS – Activity	Moderate (0.3 ≤ |rho| ≤ 0.5)	0.36	Confirmed
10. HADS – Anxiety	Moderate (0.3 ≤ |rho| ≤ 0.5)	0.39	Confirmed
11. HADS – Depression	Weak (0.1 ≤ |rho| ≤ 0.3)	0.25	Confirmed
12. NHP – Energy level	Moderate (0.3 ≤ |rho| ≤ 0.5)	0.45	Confirmed
13. NHP – Pain	Moderate (0.3 ≤ |rho| ≤ 0.5)	0.36	Confirmed
14. NHP – Emotional reaction	Moderate (0.3 ≤ |rho| ≤ 0.5)	0.35	Confirmed
15. NHP – Social isolation	Moderate (0.3 ≤ |rho| ≤ 0.5)	0.36	Confirmed
16. NHP – Sleep	Moderate (0.3 ≤ |rho| ≤ 0.5)	0.32	Confirmed
17. NHP – Physical abilities	Moderate (0.3 ≤ |rho| ≤ 0.5)	0.44	Confirmed

Statistically significant correlations are shown in bold. Confirmed hypotheses for the MDP-Resting, Immediate Perception = 17/17 (100%). MDP: Multidimensional Dyspnea Profile; VAS: Visual Analogue Scale; BMI: body mass index; MBS: Modified Borg Scale; mMRC: Modified Medical Research Council; HADS: Hospital Anxiety and Depression Scale; NHP: Nottingham Health Profile.

**Table 6:** Hypothesis testing to assess the construct validity of the MDP-Resting, Emotional Response.

Hypothesis	Construct validity is confirmed when the correlation is	Results	Interpretation of results
1. BMI	Negligible/nonsignificant(|rho| ≤ 0.1; P > 0.05)	0.07	Confirmed
2. FEV1/FVC % predicted	Nonsignificant (P > 0.05) or Weak(0.1 ≤ |rho| ≤ 0.3)	0.29	Confirmed
3. FEV1 % predicted	Nonsignificant (P > 0.05) or Weak(0.1 ≤ |rho| ≤ 0.3)	0.03	Confirmed
4. FVC % predicted	Nonsignificant (P > 0.05) or Weak(0.1 ≤ |rho| ≤ 0.3)	–0.12	Confirmed
5. VAS – Resting	Strong (|rho| ≥ 0.5)	0.59	Confirmed
6. VAS – Activity	Moderate (0.3 ≤ |rho| ≤ 0.5)	0.45	Confirmed
7. mMRC Scale	Moderate (0.3 ≤ |rho| ≤ 0.5)	0.29	Confirmed
8. MBS – Resting	Strong (|rho| ≥ 0.5)	0.43	Not confirmed
9. MBS – Activity	Moderate (0.3 ≤ |rho| ≤ 0.5)	0.24	Not confirmed
10. HADS – Anxiety	Moderate to strong (|rho| ≥ 0.3)	0.35	Confirmed
11. HADS – Depression	Moderate to strong (|rho| ≥ 0.3)	0.15	Not confirmed
12. NHP – Energy level	Moderate (0.3 ≤ |rho| ≤ 0.5)	0.39	Confirmed
13. NHP – Pain	Moderate (0.3 ≤ |rho| ≤ 0.5)	0.39	Confirmed
14. NHP – Emotional reaction	Moderate (0.3 ≤ |rho| ≤ 0.5)	0.35	Confirmed
15. NHP – Social isolation	Moderate (0.3 ≤ |rho| ≤ 0.5)	0.36	Confirmed
16. NHP – Sleep	Moderate (0.3 ≤ |rho| ≤ 0.5)	0.29	Confirmed
17. NHP – Physical abilities	Moderate (0.3 ≤ |rho| ≤ 0.5)	0.37	Confirmed

Statistically significant correlations are shown in bold. BMI: body mass index; MDP: Multidimensional Dyspnea Profile; VAS: Visual Analogue Scale; MBS: Modified Borg Scale; mMRC: Modified Medical Research Council; HADS: Hospital Anxiety and Depression Scale; NHP: Nottingham Health Profile.

**Table:** Correlations between the MDP–Activity and MDP–Resting subscales and validation variables.

	MDP–Activity		MDP–Resting	
	Immediate Perception	Emotional Response	Immediate Perception	Emotional Response
BMI, kg/m2	0.02	0.13	0.01	0.07
FEV1/FVC % predicted (n = 131)	–0.03	0.09	0.19	0.29
FEV1 % predicted (n = 136)	–0.19	–0.09	–0.03	0.03
FVC % predicted (n = 138)	–0.24	–0.18	–0.06	–0.12
VAS – Resting, mm	0.48	0.36	0.75	0.59
VAS – Activity, mm	0.68	0.59	0.48	0.45
MBS – Resting	0.41	0.35	0.60	0.43
MBS – Activity	0.59	0.39	0.36	0.24
mMRC Scale	0.38	0.30	0.31	0.29
HADS – Anxiety	0.37	0.53	0.39	0.35
HADS – Depression	0.26	0.34	0.25	0.15
NHP – Energy level	0.43	0.42	0.45	0.39
NHP – Pain	0.44	0.44	0.36	0.39
NHP – Emotional reaction	0.37	0.50	0.35	0.35
NHP – Social isolation	0.37	0.42	0.36	0.36
NHP – Sleep	0.37	0.34	0.32	0.29
NHP – Physical abilities	0.51	0.50	0.44	0.37

Statistically significant correlations are shown in bold. BMI: body mass index; MDP: Multidimensional Dyspnea Profile; VAS: Visual Analogue Scale; MBS: Modified Borg Scale; mMRC:mModified Medical Research Council; HADS: Hospital Anxiety and Depression Scale; NHP: Nottingham Health Profile.

## 4. Discussion

The current study was conducted to translate and culturally adapt the MDP into Turkish and evaluate its psychometric properties in outpatients across a range of important respiratory diseases. The Turkish version of the MDP showed excellent internal consistency and good construct validity. The test-retest reliability was excellent for 1 h. However, it tends to decrease after 1 week, especially for MDP-Resting. The explanatory factor analysis modeling demonstrated that the Turkish version of the MDP has a 2-factor structure including IP and ER.

The 2 domains of the Turkish version of the MDP showed excellent internal consistency for both Activity and Resting situations. This result was similar to that reported in previous studies. Internal consistency of these 2 domains was also moderate to very high in the Swedish [16], Portuguese [15], and English [5,11] versions of the MDP.

The Turkish version of the MDP showed excellent test-retest reliability for 1 h. However, test-retest reliability decreased moderate-to-high after 1 week. High test-retest reliability results for a short interval (hours to days) have been reported in the other validation studies [5,11,15,16]. Similar to our findings, test-retest reliability tends to decrease after weeks [5,11,16]. However, it should be noted that the decline is not dramatic, and the test-retest reliability is still at least moderate.

In this study, the MDP showed a 2-factor structure for both determined situations (i.e., activity and rest) similar to findings in the previous validation studies [5,11,15,16,18]. In the original development study of the MDP, three domains were proposed as an immediate sensory response, immediate unpleasantness, and resultant emotional response under sensory and affective dimensions based on a well-developed conceptual model of pain perception [3]. In that model, the “breathing discomfort” item should be in the affective dimension. However, our study and other studies suggested that the MDP has a 2-factor structure as IP and ER domains, and “breathing discomfort” item is clustered under the IP domain [5,11,12,15,16,18]. These results suggest that 2 domains are necessary to describe dyspnea.

In our study, most of the predetermined hypotheses were confirmed showing construct validity. Although some outcome measures were different from ours and some correlations were slightly different for the same measures in the other validation studies, they also reported that the MDP had construct validity [5,11,15,16,18]. Since the FEV1 is weakly associated with the severity of dyspnea [18], we expected to find nonsignificant or weak correlations and it was confirmed. Ekström et al. [16], Williams et al. [18], and Meek et al. [5] also showed weak correlations between the MDP domains and FEV1 results. In addition to the FEV1, we also investigated the other commonly used spirometry parameters (FEV1/FVC and FVC), and the correlation results were quite similar as no significant or weak correlations were observed. Meek et al. [5] also reported no significant correlation between the MDP domains and FVC, and Williams et al. [18] reported no significant or weak correlations between the FEV1/FVC and MDP domains. These results extend the evidence that spirometry results were weakly associated with the perception of dyspnea. To further support the divergent validity of the MDP, we investigated the correlations with BMI and found no significant correlation per Meek et al. [5].

Based on the underlying concept of MDP, many studies evaluated depression and anxiety levels to assess its concurrent validity. Strong correlations between the MDP domains and HADS-Anxiety and HADS-Depression were observed in these studies [15,16,18] as in our results. Although Meek et al. [5] used different outcome measures (Brief Symptom Inventory) to assess depression and anxiety, they also showed strong correlations.

Many studies also used the mMRC scale, which assesses functional impairment due to dyspnea to investigate the validity of the MDP and reported moderate to strong correlations [5,15,16]. We also observed moderate correlations between the MDP domains and the mMRC scale. To further support the concurrent validity, we also used the MBS and VAS. Since we used the same periods and situations while administrating the MBS and VAS, their correlations were much stronger compared to the mMRC scale. Williams et al. [18] and Banzett et al. [12] also used the mMRC, VAS, and MBS, and similarly and found similar results. Since the mMRC has embedded questions whereas the VAS and MBS asked the same time frame and situations as the MDP, these results were expected.

It is a well-known fact that dyspnea can be associated with poor health-related quality of life [28]. Therefore, we included a health-related quality of life measure as an anchor to investigate the concurrent validity of MDP and found moderate to strong correlations between the MDP and subdomains of health-related quality of life measures. Only the Swedish validation study of the MDP used a quality of life measure and found similar results [16]. These findings suggest that dyspnea is closely associated with health-related quality of life, not only in a unidimensional [28] but also in a multidimensional manner.

Since the MDP is not a disease-specific measure, the MDP validation studies were conducted under different conditions, and laboratory and clinical settings included patients with dyspnea in the acute emergency setting, patients with COPD, or outpatients with various cardiorespiratory diseases [4,5,11,12,15,16,18,29,30]. We also investigated the psychometric properties of the MDP in a quite different population including outpatients with different respiratory diseases, and found similar reliability and validity results. These findings support that the MDP is valid for measurement and comparison of dyspnea across different populations and settings. Therefore, the Turkish version of the MDP can be used without adaptations in various conditions and settings.

Another advantage of the MDP is that the time frame and situation can be specified by the user, depending on the setting and aim [12]. The validation studies used many different periods and situations including “during the last 2 weeks”, “during the last 15 days”, “right now”, “now”, “the worst experience”, “during activities of daily living”, “on average over the past 2 weeks”, “when you decided to come to the emergency department”, and “during the last minute of the walk test” [5,11,13,15,18]. In this study, we used time frames and situations such as “while resting without doing any physical activity during the past 2 weeks” and “at the end of climbing a flight of 2-floor stairs during the past 2 weeks”. We intended to focus on a specific activity that is known to trigger dyspnea, and a normal resting state. Similar findings obtained from different studies suggest that the psychometric properties of MDP are consistent across different time frames and situations, even if the actual intensity levels for items may vary.

Our study had some limitations. First, we did not investigate other psychometric properties such as responsiveness and interrater reliability. However, Meek et al. [5] showed that the responsiveness to change in MDP domains with treatment and Belo at al. [15] found that the MDP was reliable independently of different raters. Second, we performed a principal component analysis rather than a confirmatory factor analysis. However, both methods generally agree on the number of components and which items load primarily on which factors [31]. Supporting this, some MDP validation studies used a principal component analysis and the others used a confirmatory factor analysis or both, and their results are consistent [5,11,12,15,16,18]. Another limitation is the use of a convenience sample and the exclusion of the patients who did not speak or understand Turkish, who were unwilling to participate, and who had completed the study measures.

Apart from the limitations noted above, our study had several strengths. Our sample was large and included outpatients with many different respiratory diseases, supporting the existing evidence that the MDP is not a disease-specific instrument. Our study demonstrated for the first time that the MDP is valid and reliable to assess the dyspnea in a resting state without physical activity. Apart from many previous validation studies of MDP, we used a relatively broad range of outcome measures to assess validity. Considering the high prevalence of Turkish-speaking populations not only in Turkey but also in Europe, we believe that the Turkish version of the MDP will gain a high utilization rate. The MDP has been validation in many languages and many other language validations studies are ongoing that show its international acceptance. The Turkish version of the MDP will allow conducting multicultural and multicentered international studies.

In conclusion, the MDP was successfully translated and culturally adapted into Turkish, and this version showed good psychometric properties including the factorial structure, internal consistency, test-retest reliability, and construct validity to assess multidimensional aspects of dyspnea.

## Informed Consent

The study was approved by the Noninvasive Research Ethics Board of Dokuz Eylül University (approval number: 2018/18-40 and date: 19.07.2018) and performed following the ethical standards as laid down in the 1964 Declaration of Helsinki (as revised in Brazil 2013). All the participants gave written informed consent before participation in the study.
